# Penetrating eye injury by dart

**DOI:** 10.1007/s00414-020-02477-9

**Published:** 2020-12-17

**Authors:** Tanja Germerott, N. Mann, S. Axmann

**Affiliations:** Institute of Legal Medicine, University Medical Center of the Johannes Gutenberg University Mainz, Am Pulverturm 3, 55131 Mainz, Germany

**Keywords:** Dart, Eye injury, Eye bulb injury, Penetrating eye injury, Foreign body

## Abstract

Darts are constantly gaining in popularity. However, their risk of injury is often underestimated. This report is about a juvenile who suffered from a severe eye injury including the opening of the eye bulb. The attending ophthalmologists ruled out the possibility that this kind of injury could be caused by a dart with a plastic point. However, by reconstructing the course of action and throwing darts at porcine eyes, the forensic medical advisory opinion was able to state that darts with damaged plastic points may cause the exact same form of injury. This casuistic illustrates the essential significance of forensic-traumatological knowledge and, especially in the case of rare injury patterns, case-related practical experiments.

## Introduction

The game of darts originated in the early 20th century. It is constantly increasing in popularity not only for leisure activities but also for highly endowed competitive sports [1]. Every dart has four parts: the point (or tip), barrel, shaft, and flight [2]. In medical literature, there are only few casuistic reports or case series of eye injuries caused by darts. In the majority of these cases the injuries were caused by the points, which come in plastic or steel versions, or the flights [3–5]. In general, darts bear a relevant risk of injury if used carelessly or improperly.

Children and juveniles are the most highly endangered age groups for suffering an eye injury. About 25% of all penetrating eye injuries can be found in these age groups, males predominating over females [3, 6–8].

The report below presents a rare case of a perforating eye injury caused by the plastic point of a dart.

## Casuistic

A 16-year-old female juvenile underwent eye surgery after having suffered a perforating scleral bulb injury along the limb with a prolapse of iris tissue, ranging between 12 and 4 o’clock according to the clinical reports. There were no further injuries, especially facial or around the eyes, documented in the medical records. Prior to surgical treatment, no photography was taken. After the juvenile’s discharge from hospital, her visual acuity constituted 1%.

According to police inquiry, the injured person had visited a Christmas market and sat on a metal fence. Nearby, three juveniles had fooled around with a plastic-tipped dart, which they had stolen from a bar. Supposedly the dart had been purposefully thrown to the ground to make it stick in the soil. Finally, one of the juveniles had thrown the dart nondirectional to the side in order to prevent harm. From the corner of her eye, the injured person had seen something flying towards her, followed by a sudden pain in the eye. Afterwards, the dart had fallen to the ground. When interrogated, she negated having tumbled. The dart had been unable to seize.

During the following trial, two ophthalmologists from the treating hospital testified that the pattern of injury described above could not be induced by a dart’s point, stating that in this case the injury would have to be punctual. Furthermore, they testified that in this particular case something would have to hit the eye with 100 km/h and that the injury in question seemed to have been caused by falling on the edge of a table, regardless of the juvenile’s statement that she had not tumbled.

**Fig. 1:**
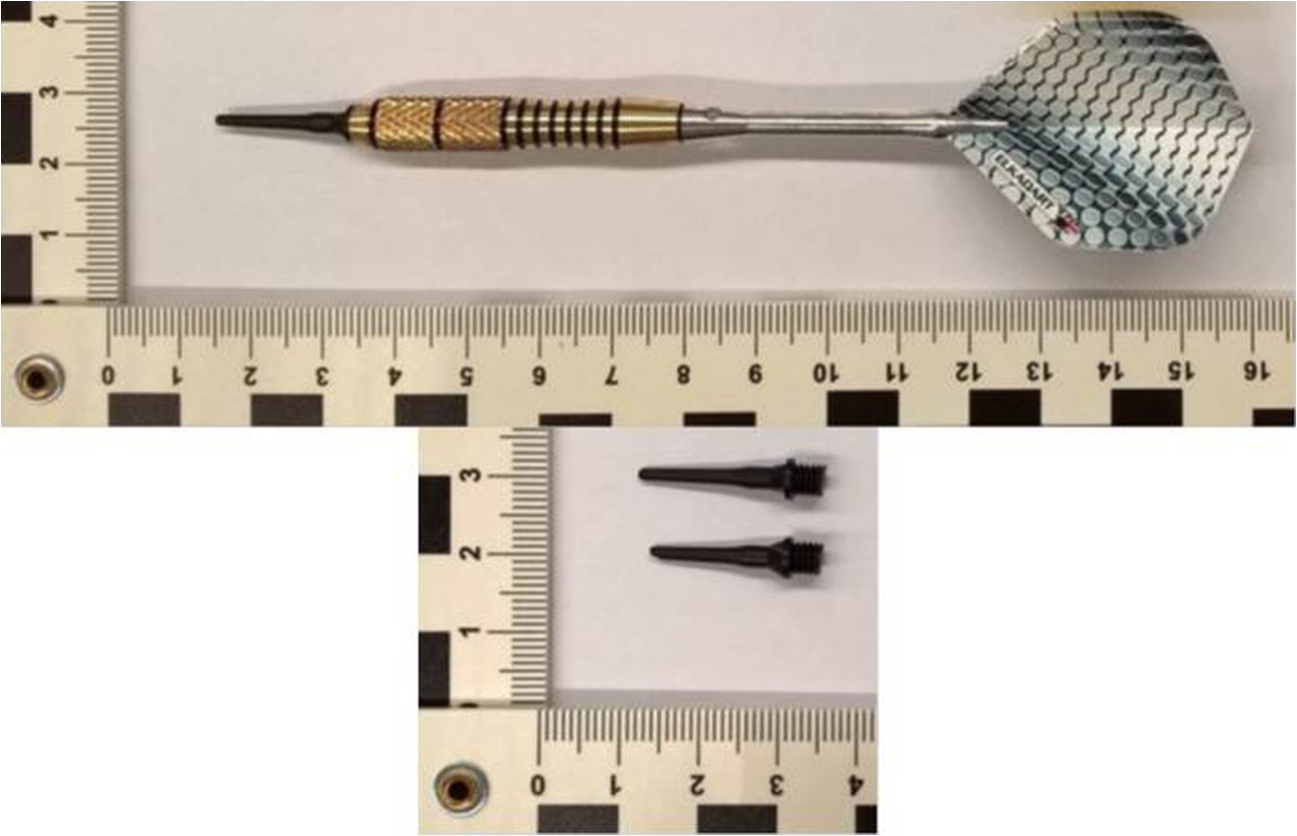
**a** Dart and plastic points. The upper one unscathed the one down below with a modified plastic point. **b** Injury to the pig’s eye (red arrow)

**Fig. 2:**
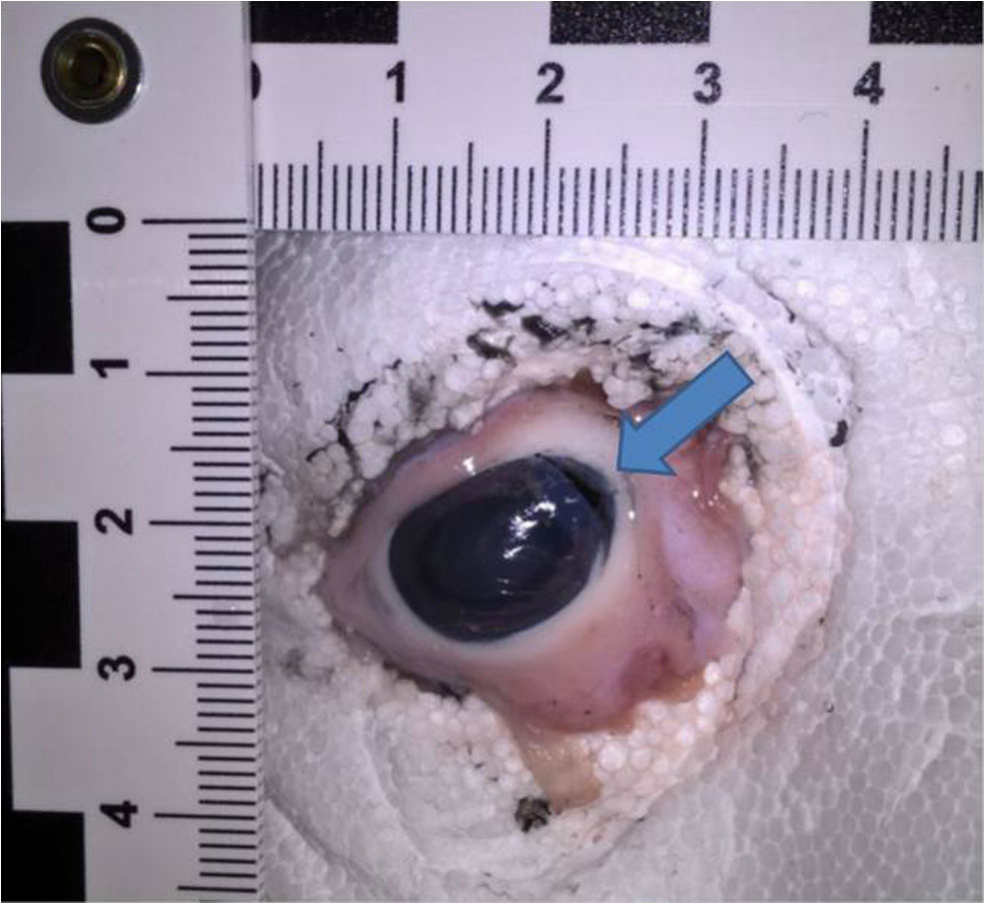
An injury very similar to the juvenile’s, induced by a dart with a pinched-off point

Eventually, a forensic medicine’s advisory opinion was obtained. Darts were first dropped on the eyes from a height of 10 m using a drop tube. The average speed of the darts was determined by means of time measurements (Table 1). The series of tests have shown that darts with metal tips can result in serious injuries and perforations of the eyeball. Darts with undamaged plastic tips caused only slight visible, isolated injuries to the cornea. Darts with three kinds of points were then thrown at porcine eyes for information assessment: steel points, plastic points, and modified plastic points, simulating the damage of the dart point described above by pinching it off (Fig. 1). The darts were thrown by hand at the porcine eyes, which were embedded in styrofoam for fixation. Darts were thrown by hand over 50 times, but only few darts hit the porcine eye (no experience in darts was present). But finally after one throw, injury very similar to the juvenile’s was induced by a dart with a pinched-off point (Fig. 2).

**Table 1:** List of test results with hand-thrown darts at a distance of 2 m

Throw no.	Throw distance *d* (m)	Travel time *t* (s)	Average speed^*^ (m/s)
1	2	0.19	10.53
2	2	0.20	10.00
3	2	0.19	10.53
4	2	0.19	10.53
5	2	0.21	9.52
6	2	0.20	10.00
7	2	0.19	10.53
8	2	0.21	9.52
9	2	0.19	10.53
10	2	0.20	10.00

^*^Average speed rounded two decimal places

The kinetic energy of the points added up to 0.84 J in throws from a 5-m distance. The flight of the dart did not result in an opening of the bulb, neither by throwing it nor by cutting the porcine eye with it. There were only superficial corneal lesions definable.

In the end, the charge was dismissed due to diverging testimony and the missing evidence weapon.

## Discussion

The incidence of treatment-demanding eye injuries amounts to 810/100,000 inhabitants [9]. The high number of eye injuries is not just a socio-economic issue but also one of the main causes of amaurosis [10–12]. By introducing a mandatory seatbelt in England in 1983, perforating eye injuries were lowered by 11.1%. This underlines the impact of simple primary preventional actions on the incidence of eye injuries [6]. In literature, there are various cases of partly perforating eye injuries caused by diverse objects such as durian fruit, fishing gear, or darts [3, 4, 13, 14]. The rather rare eye injuries caused by darts mostly lead to a fulminant reduction of visual acuity and lots of after treatment due to persistent vitreous body bleedings [3, 4]. These dramatic etiopathologies and the high potential of injury should be kept in mind when handling darts. There are over-the-counter soft-air guns whose projectiles generate a kinetic energy from 0.5 J up to 7.5 J [15, 16]. These “toy” guns carry an immense risk of injury, considering that corneal lesions occur at 0.184 J already [17]. Soft air guns subject to weapons law when generating a kinetic energy of ≥ 0.5 J, resulting in strict terms of carrying and using them [16]. In the present case, the kinetic energy of a thrown dart added up to 0.84 J, which is comparable to the lower kinetic energy of soft air guns.

In this particular case, a forensic medicine’s opinion was obtained only due to the conflicting testimony of the ophthalmologists and the injured person. Especially in scarce patterns of injury, a forensic-traumatologic expertise is mandatory for information evaluation and reconstruction, which in this case proved that falling on a table’s edge when there were no other injuries on exposed body parts cannot have been causal for the described eye injury.

Since dart-related injuries are rare, the present case illustrates the essential significance of case-related practical experiments as an essential element of forensic medicine. Against the ophthalmologists’ statement, they were able to prove that a pinched-off darts point may very well cause a bulb injury comparable to the one described.
